# Improving paediatric asthma care in Zambia

**DOI:** 10.2471/BLT.14.144071

**Published:** 2015-08-19

**Authors:** Somwe Wa Somwe, Emilia Jumbe-Marsden, Kondwelani Mateyo, Mutale Nsakashalo Senkwe, Maria Sotomayor-Ruiz, John Musuku, Joan B Soriano, Julio Ancochea, Mark C Fishman

**Affiliations:** a Department of Paediatrics and Child Health, University Teaching Hospital, P/Bag RW 1X, Lusaka, Zambia.; b Pendleton Family Practice, Lusaka, Zambia.; c University Teaching Hospital, Lusaka, Zambia.; d Ministry of Health, Lusaka, Zambia.; e Sandoz Kenya, Nairobi, Kenya.; f IdISPa-FISIB, Hospital Universitari Son Espases, Palma de Mallorca, Illes Balears, Spain.; g Hospital de la Princesa, Madrid, Spain.; h Novartis Institutes for BioMedical Research, Cambridge, United States of America.

## Abstract

**Problem:**

In 2008, the prevalence of paediatric asthma in Zambia was unknown and the national treatment guideline was outdated.

**Approach:**

We created an international partnership between Zambian clinicians, the Zambian Government and a pharmaceutical company to address shortcomings in asthma treatment. We did two studies, one to estimate prevalence in the capital of Lusaka and one to assess attitudes and practices of patients. Based on the information obtained, we educated health workers and the public. The information from the studies was also used to modernize government policy for paediatric asthma management.

**Local setting:**

The health-care system in Zambia is primarily focused on acute care delivery with a focus on infectious diseases. Comprehensive services for noncommunicable diseases are lacking. Asthma management relies on treatment of acute exacerbations instead of disease control.

**Relevant changes:**

Seven percent of children surveyed had asthma (255/3911). Of the 120 patients interviewed, most (82/120, 68%) used oral short-acting β_2_-agonists for symptom control; almost half (59/120, 49%) did not think the symptoms were preventable and 43% (52/120) thought inhalers were addictive. These misconceptions informed broad-based educational programmes. We used a train-the-trainer model to educate health-care workers and ran public awareness campaigns. Access to inhalers was increased and the Zambian standard treatment guideline for paediatric asthma was revised to include steroid inhalers as a control treatment.

**Lessons learnt:**

Joint activities were required to change paediatric asthma care in Zambia. Success will depend on local sustainability, and it may be necessary to shift resources to mirror the disease burden.

## Résumé

**Problème:**

En 2008, la prévalence de l'asthme chez les enfants en Zambie était inconnue et les recommandations nationales en matière de traitement étaient obsolètes.

**Approche:**

Nous avons mis en place un partenariat international entre des médecins zambiens, le gouvernement du pays et une société pharmaceutique afin de remédier aux manques dans le traitement de l'asthme. Nous avons mené deux études, une pour estimer la prévalence de la maladie dans la capitale, Lusaka, et l'autre pour évaluer les comportements et les pratiques des patients. Les informations obtenues nous ont permis de sensibiliser les professionnels de santé ainsi que le grand public. Elles ont également contribué à moderniser la politique du gouvernement concernant la prise en charge pédiatrique de l'asthme.

**Environnement local:**

Le système de santé en Zambie est essentiellement axé sur la prestation de soins de courte durée, notamment pour traiter les maladies infectieuses. Il n'offre pas de services complets pour le traitement des maladies non transmissibles. La prise en charge de l'asthme repose sur le traitement des exacerbations aigües plutôt que sur le contrôle de la maladie.

**Changements significatifs:**

Sept pour cent des enfants de l'étude avaient de l'asthme (255/3911). Sur les 120 patients interrogés, la plupart (82/120, 68%) utilisaient des β_2_-stimulants à action brève par voie orale pour soulager les symptômes; près de la moitié (59/120, 49%) ne pensaient pas que les symptômes étaient évitables et 43% (52/120) pensaient que les inhalateurs créaient une dépendance. Ces idées fausses ont orienté la conception de vastes programmes de sensibilisation. Nous avons utilisé un modèle de formation des formateurs à l'intention des professionnels de santé et mené des campagnes de sensibilisation à destination du public. L'accès aux inhalateurs a été élargi et les recommandations de la Zambie quant au traitement standard de l'asthme ont été révisées afin d'inclure les inhalateurs de stéroïdes comme traitement de fond.

**Leçons tirées:**

Des activités conjointes ont été requises pour modifier la prise en charge pédiatrique de l'asthme en Zambie. Leur succès dépendra de leur durabilité au niveau local; il pourra aussi être nécessaire de réaffecter des ressources en fonction de la charge de morbidité.

## Resumen

**Situación:**

En 2008, se desconocía la prevalencia del asma infantil en Zambia y las directrices nacionales sobre el tratamiento estaban obsoletas.

**Enfoque:**

Con el objetivo de poner remedio a las deficiencias en el tratamiento del asma, se creó una alianza internacional entre los médicos zambianos, el Gobierno de Zambia y una compañía farmacéutica. Se llevaron a cabo dos estudios, uno para obtener una estimación de la prevalencia en Lusaka, la capital, y otro para evaluar los comportamientos y consultas con los pacientes. En base a la información obtenida, se educó tanto al personal sanitario como al público general. La información obtenida de los estudios también se utilizó para modernizar la política del gobierno sobre la gestión del asma infantil.

**Marco regional:**

El sistema sanitario de Zambia se centra principalmente en una rigurosa prestación de asistencia sanitaria, con el punto de mira puesto en las enfermedades infecciosas. Hay una falta de servicios integrales para las enfermedades no contagiosas. La gestión del asma radica en el tratamiento de la exacerbación aguda en lugar del control de la enfermedad.

**Cambios importantes:**

El siete por ciento de los niños encuestados han tenido asma (255/3.911). De los 120 pacientes entrevistados, la mayoría (82/120, un 68%) utilizaron agonistas β_2_ de acción corta por vía oral para controlar los síntomas; casi la mitad (59/120, un 49%) consideraron que los síntomas no se podían prevenir y un 43% (52/120) pensaron que los inhaladores eran adictivos. Estas ideas equivocadas sirvieron para informar a los programas educativos de amplio espectro. Se utilizó un modelo para formar a los instructores para educar al personal sanitario y se llevaron a cabo campañas de sensibilización de los ciudadanos. Aumentó el acceso a los inhaladores y se revisaron los estándares de las directrices sobre el tratamiento del asma infantil en Zambia para incluir esteroides inhalados como parte del tratamiento de control.

**Lecciones aprendidas:**

Hizo falta unir fuerzas para cambiar el cuidado del asma infantil en Zambia. El éxito dependerá de la sostenibilidad del lugar y puede que sea necesario hacer un intercambio de recursos para así poder reflejar la carga de la enfermedad.

## ملخص

**المشكلة:**

لم تتوفر أي معلومات عن انتشار الربو لدى الأطفال في زامبيا في عام 2008 وكان المبدأ التوجيهي القومي بشأن العلاج لا يعكس المتطلبات الحديثة.

**الأسلوب:**

أقمنا علاقة شراكة دولية بين الأطباء المعالجين في زامبيا وحكومة زامبيا، وإحدى شركات صناعة الأدوية لتعويض القصور في علاج الربو. وأجرينا دراستين، تناولت إحداهما الانتشار التقديري للمرض في العاصمة لوساكا، بينما تناولت الأخرى تقييم اتجاهات المرضى وممارساتهم. ووفقًا للمعلومات التي حصلنا عليها، عملنا على تثقيف العاملين بمجال الخدمات الصحية وعامة الناس في هذا الصدد. كما استخدمنا المعلومات التي تضمنتها هاتان الدراستان لتحديث سياسة الحكومة في التعامل مع مرض الربو لدى الأطفال.

**المواقع المحلية:**

ينصب الاهتمام في نظام الرعاية الصحية بزامبيا في المقام الأول على تقديم الرعاية الصحية للحالات التي تعاني بشدة من المرض مع التركيز على الأمراض المعدية. وبالإضافة إلى ذلك فإن هناك غياب للخدمات الشاملة المقدمة لحالات الأمراض غير المعدية. ويعتمد التعامل مع مرض الربو على علاج الحالات الحادة المتدهورة بدلًا من السيطرة على المرض.

**التغيّرات ذات الصلة:**

كان سبعة بالمائة من الأطفال ممن شملهم المسح يعانون من الربو (255/3911). واستخدم معظم المرضى (82.68%) من بين 120 مريضاً تمت مقابلتهم ناهضات بيتا 2 قصيرة المفعول للسيطرة على الأعراض؛ ورأي نصفهم تقريبًا (59.49%) أنه لا يمكن منع المعاناة من الأعراض، بينما ظن 43% (52/120) منهم أن البخاخات تسبب الإدمان. ووفرت هذه المفاهيم المغلوطة المعلومات اللازمة للبرامج التثقيفية العامة. واستخدمنا نموذج تدريب المدربين لتثقيف العاملين بمجال تقديم الخدمات الصحية وبدأنا في نشر حملات عامة للتوعية. وزاد معدل استخدام البخاخات وتمت مراجعة المبدأ التوجيهي المعياري لعلاج الربو لدى الأطفال بزامبيا، حيث تضمن استعمال بخاخات الستيرويدات كعلاج للسيطرة على المرض.

**الدروس المستفادة:**

اقتضت الحاجة اللجوء إلى الأنشطة المشتركة للتغيير من الرعاية الصحية المقدمة لحالات الربو لدى الأطفال في زامبيا. وسيتوقف النجاح في ذلك على الاستدامة المحلية، وقد تدعو الضرورة إلى تبديل المصادر بما يعكس عبء المرض.

## 摘要

**问题:**

在 2008 年，儿童哮喘在赞比亚的盛行原因不明，当时全国的治疗指南不合时宜。

**方法:**

我们与赞比亚的临床医生、赞比亚政府和一家制药公司建立了合作关系，共同找出哮喘治疗中的不足之处。 我们进行了两项研究，一项是估量首都卢萨卡的患病率，另一项是评估患者的态度和做法。 我们以获得的信息为基础，对医务工作者和公众进行教育。 在儿童哮喘管理方面，还采用了研究所得的信息，以适应政府政策的现代化需求。

**当地状况:**

赞比亚的医疗系统主要力量集中在急性医疗服务，重点在传染性疾病。 对非传染性疾病的综合服务较缺乏。 哮喘管理依赖于急性发作治疗，而不是疾病控制治疗。

**相关变化:**

被调查的儿童中，有 7% (255/3911) 的人患有哮喘。 在采访的 120 名患者中，大多数 (82/120,68%) 患者用口服短效 β_2_ 受体激动剂来控制症状，几乎有一半 (59/120,49%) 患者认为症状是不可预防的，43% 的患者(52/120) 认为使用吸入器会上瘾。 这些错误的想法表明需要实行广泛的教育计划。 我们用培训教员的模式来教育医务工作者，还举行了多项宣传活动，以提高公众意识。 现在吸入器的使用者增多了，有关赞比亚儿童哮喘标准的治疗指南也进行了修订，将类固醇吸入器纳入控制治疗中。

**经验教训:**

需要各方共同合作来改变赞比亚的儿童哮喘医疗状况。 可持续性发展是取得成功的重要因素，并且可能有必要通过转移资源来减轻疾病负担。****

## Резюме

**Проблема:**

По состоянию на 2008 год данные по распространенности заболеваний астмой у детей в Замбии отсутствовали. Национальные рекомендации по ее лечению устарели.

**Подход:**

Мы сформировали международную группу, в которую привлекли замбийских врачей, правительство Замбии и одну фармацевтическую компанию, для устранения недостатков, связанных с лечением астмы. Нами были проведены два исследования: одно для оценки распространенности заболевания в столице, г. Лусака, и второе для оценки того, какого поведения и каких практических мер придерживаются пациенты. Опираясь на полученные сведения, мы организовали обучение медицинских работников и представителей общественности. Информация, полученная в ходе исследований, была также использована для модернизации правительственной политики в области лечения астмы у детей.

**Местные условия:**

Система здравоохранения в Замбии в первую очередь ориентирована на лечение острых случаев, при этом особое внимание уделяется инфекционным заболеваниям. Ощущается недостаток комплексного обслуживания в тех случаях, когда пациент страдает неинфекционным заболеванием. В лечении астмы врачи опираются на купирование случаев обострения, но не ориентированы на контроль заболевания.

**Осуществленные перемены:**

Семь процентов из числа обследованных нами детей (255 из 3911) страдали от астмы. Из 120 опрошенных пациентов большинство (82 человека, 68%) использовали пероральные β_2_-агонисты краткосрочного действия для подавления симптомов, почти половина (59 человек, 49%) не думали, что симптомы можно предотвратить, и 43% (52 из 120 человек) полагали, что ингаляторы вызывают привыкание. Такие неправильные представления возвели в принцип в образовательных программах для общественности. Мы использовали модель «обучи учителя» для того, чтобы научить медицинских работников и запустить кампанию по информированию общественности. Доступ к ингаляторам расширился, и в стандартные замбийские рекомендации по лечению астмы у детей после их пересмотра были включены ингаляторы со стероидами в качестве контрольного вида лечения.

**Выводы:**

Потребовались совместные усилия для того, чтобы изменить характер лечения детской астмы в Замбии. Успех будет зависеть от стабильности ситуации на местах, и может потребоваться перераспределение ресурсов для лучшего контроля за распространенностью болезни.

## Introduction

Asthma is the most common paediatric chronic disease.^1^ In 2006, approximately 14% of the world’s children experienced asthma symptoms.^2^^,^^3^ In African countries, the prevalence of asthma ranges from approximately 10% to more than 20%.^2^^–^^4^ Poorly treated asthma can lead to school absence, hospitalization and death.^5^ Yet, effective medical management of patients with asthma is available. During acute exacerbations, inhalation of short-acting β_2_-agonists is recommended to provide relief. Long-term control of the disease is normally achieved using inhaled steroids, but long-acting β_2_-agonists, oral leukotriene modifiers or injectable anti-immunoglobulin E antibodies are also used in more severe cases.^6^^,^^7^

The paediatric clinic of the main teaching hospital in Zambia often sees children with severe asthma. However, these children are rarely diagnosed as having asthma and most have not been treated with β_2_-agonists or steroid inhalers. Until recently, asthma inhalers have not been readily available in the country, in part because the national guideline preferentially endorsed oral and intravenous treatments for asthmatic children (Box 1).^8^ When inhalers were offered, patients were often reluctant to use them because of misconceptions about efficacy and addiction.

Box 1 **Zambia standard treatment guidelines for paediatric asthma before and after the activities of the public–private partnership**

**Guideline before 2008^8^**

Management instructions for the asthmatic child.

- Child with wheezing but no rapid breathing: oral salbutamol.
- Child with wheezing and respiration rate of over 50 per minute: aerosolized salbutamol using nebulizer and enriched air with oxygen, followed by oral salbutamol.
- Very ill child: nebulized salbutamol plus intravenous aminophylline, intravenous hydrocortisone, and oral prednisolone.

**Guideline after 2013^9^**

Management instructions for children aged 5–12 years: step up to improve control as needed and step down to find and maintain the lowest controlling step; patients should start treatment at the step most appropriate to the initial severity of their asthma; check concordance and reconsider diagnosis if response to treatment is unexpectedly poor.

- Step 1 – mild intermittent asthma: inhaled short-acting β_2_-agonist as required.
- Step 2 – regular preventive therapy: add inhaled steroid (or other preventer if inhaled steroid cannot be used); start at dose of inhaled steroid appropriate to severity of disease.
- Step 3 – initial add-on therapy: first, add inhaled long-acting β_2_-agonist (LABA). Second, assess control of asthma. Good response to LABA: continue LABA. Benefit from LABA but control still inadequate: continue LABA and increase inhaled steroid dose. No response to LABA: stop LABA and increase inhaled steroid dose; if control still inadequate, institute trial of other therapies, leukotriene receptor antagonist or theophylline.
- Step 4 – persistent poor control: increase inhaled steroid dose.
- Step 5 – continuous or frequent use of oral steroids: use daily oral steroid tablet in lowest dose providing adequate control. Maintain high dose inhaled steroid. Refer to respiratory paediatrician.

The prevalence of paediatric asthma in Zambia is unknown and there is a poor understanding of disease progression and management on the part of patients, families and health-care providers. Hence, we were faced with the complex problem of disease recognition, misconceptions about diagnosis and therapy and poor access to asthma medicines.

## Local setting

The health-care system in Zambia is primarily focused on acute care delivery with a particular focus on infectious diseases. Comprehensive services for noncommunicable diseases are lacking. Asthma management relies on the treatment of acute exacerbations instead of disease control.^8^

## International partnership

To address the problem of paediatric asthma, we formed an international public–private partnership involving clinicians at the Lusaka University Teaching Hospital, officials from the noncommunicable diseases unit at the Zambian Ministry of Health, the Spanish Society of Pneumology and Thoracic Surgery, the International Center for Advanced Respiratory Medicine and Novartis. Nearly a year of planning with our partners preceded the implementation of the programme’s multifaceted activities.

### Original research

The partnership conducted two epidemiological studies, one that documented paediatric prevalence and risk factors for asthma and one that characterized existing attitudes and practices of patients with the disease. The prevalence study was conducted using questionnaires from the International Study of Asthma and Allergies in Childhood (ISAAC).^10^ We used the information from the studies to improve care through training programmes for health workers, public education and government advocacy.

To estimate the asthma burden in Lusaka, we used methods and tools developed by the ISAAC.^11^ The prevalence of asthma in children aged 7–8 years was 5% (100/2026) and that in adolescents aged 13–14 years was 8% (155/1885), similar to those measured in neighbouring African countries.^2^

To assess attitudes and practices of people with asthma, we surveyed 10 children older than nine years and 110 people older than 18 years with asthma attending four primary health-care clinics in Lusaka.^12^ Most (68%; 82/120) used oral short-acting β_2_-agonists for symptom control, while inhaled steroids were used by only 14% (17/120). Nearly 30% (35/120) did not think an inhaler is a good treatment for asthma and 43% (52/120) believed inhalers were addictive. Almost half (49%; 59/120) of the participants did not think that asthma symptoms were preventable with medications.

### Education and awareness

Two main themes emerged from the data that guided the message of our projects. First, paediatric asthma was common; therefore there was a need to improve the diagnostic and treatment skills of health workers. Second, misconceptions about asthma therapies were common. We designed programmes directed to health workers and patients to improve their understanding of the disease, correct prejudices against inhaler therapy and facilitate access to effective medicines.

Few health workers in Zambia had received specialty training in asthma management. Through a train-the-trainer model, local instructors – two paediatricians and one physician from the University Teaching Hospital and the ministry of health – trained doctors, clinical officers and nurses from Lusaka’s urban clinics and health facilities in five provinces. During a two-day course, the trainees learned current algorithms of care, spacer and inhaler technique, spirometry and use of peak-flow meters. The participants received travel reimbursement. If trainees needed advice after the course finished, they were able to contact the instructors by phone. More than 80 trainees have been trained as service providers across Zambia. They provide ongoing tutoring as well as in-service training to clinical staff.

In parallel with the in-country trainings, the three local instructors received sponsored personalized training at specialty centres in Spain.

To improve asthma awareness and care, the World Asthma Day^13^ is organized each year by the Global Initiative for Asthma, which distributes materials and resources. In 2011 and 2012, we organized the event in Zambia. Officials from the ministry of health participated as guests of honour. Zambian physicians gave radio interviews and an asthma awareness radio clip was broadcast nationwide. A theatrical group performed an original production that challenged common asthma misconceptions. Inhaler demonstrations were held, and approximately 150 people participated in a 2 km walk-for-asthma in Lusaka.

### Access to treatment

To increase access to asthma medicines and tools, Sandoz, 1A Pharma, and Sibelmed donated salbutamol (short-acting β_2_-adrenergic agonist) and beclomethasone (corticosteroid) inhalers, spacers, spirometers and peak-flow meters while our training and awareness activities were scaled up. Between 2010 and 2013, more than 1850 asthma inhalers and spacers were provided free of charge in accordance with World Health Organization guidelines for drug donations.^14^ Eleven clinics in Lusaka served as inhaler distribution points. A pharmacovigilance programme was implemented to monitor for adverse effects. Between 2010 and 2013, no adverse effects were reported.

### Changing health policy

The bottom-up approach raised awareness of paediatric asthma and enabled advocacy by the partnership’s physicians to modernize the health policy concerning disease management. The ministry of health revised the national guideline for paediatric asthma management. In accordance with international recommendations,^5^^,^^15^^,^^16^ β_2_-agonists and steroid inhalers are now used for disease control (Box 1).^9^ This achievement is anticipated to promote scale-up of improved asthma-care practices nationwide, especially via educational activities and access to inhalers across the country. Salbutamol and beclomethasone inhalers have now been included in the Zambian Essential Medicines List and are available through an autonomous government facility that stores all medicines for public health institutions. The government policy states that these medicines should be available at all public sector health facilities across Zambia. We have observed that the policy change, in concert with training and awareness activities, has improved the care of children with asthma. Urban clinics and health facilities from other parts of the country are requesting inhaled asthma medication.

## Next steps

Changes in the attitude of health workers and patients are underway at some of the larger referral hospitals. However, more work is needed in these hospitals – including refresher courses and continuous medical education for staff – and no changes have yet been implemented at peripheral health facilities, which serve most of the population. Continued investigation of patient behaviours and attitudes will help to tailor further educational messages. Out of the 10 provinces in Zambia, two are predominantly urban and the rest are rural. Assistance from international partners might be helpful in reaching remote populations in Zambia.

## Lessons learnt

The main lessons learnt are summarized in Box 2. We show that a public–private partnership to improve care for one specific paediatric chronic disease is feasible in Zambia. A combination of physicians dedicated to the specific illness, a prevalence study, the use of adequate treatment and understanding the misconceptions of patients, have improved the care of asthma. Policy changes, modernization of government regulations and better availability of medicines also contributed to the improvement. We believe that by using the same strategy, improvement of care for other common noncommunicable diseases will be possible.

Box 2 **Summary of main lessons learnt**

- Improving paediatric asthma care in Zambia is feasible.
- To improve compliance with essential asthma therapies, misconceptions about the treatment of asthma must be addressed.
- To improve the provision of paediatric asthma care in Zambia, a combination of epidemiological studies, health-worker training, access to medicine and policies that are tailored to local needs were required.

While continuing efforts will be needed to fully understand and address misconceptions relating to asthma therapy, we have anecdotally noted an increase in acceptability of inhaled medications among paediatric asthma patients and their families.

Given the epidemiological transition currently experienced in Zambia and other African countries, the improved asthma programme presented here might be funded by shifting some health-care resources from infectious to chronic diseases. The government’s continued role in advocacy, health system strengthening and assuring inhaler availability, will be needed to sustain the improved asthma care established by the new treatment guidelines.
